# Congenital Goitrous Hypothyroidism, Deafness and Iodide Organification Defect in Four Siblings: Pendred or Pseudo−Pendred Syndrome?

**DOI:** 10.4274/jcrpe.v2i2.81

**Published:** 2010-05-06

**Authors:** Cengiz Kara, Mehtap Kılıç, Ahmet Uçaktürk, Murat Aydın

**Affiliations:** 1 Ondokuz Mayıs University, Pediatric Endocrinology, Samsun, Turkey; +90 362 312 1919/3301+90 362 457 60 41cengizkara68@yahoo.comOndokuz Mayıs University, Faculty of Medicine, Departments of Pediatric Endocrinology, 55139, Kurupelit, Samsun, Turkey

**Keywords:** Pendred syndrome, pseudo−Pendred syndrome, congenital hypothyroidism, thyroid peroxidase defect

## Abstract

Pendred syndrome (PDS) is an autosomal recessive disorder characterized by congenital deafness, goiter and iodide organification defect. Presence of inner ear malformations is essential for the clinical diagnosis. Most individuals with PDS are clinically and biochemically euthyroid. Mutations in the PDS gene encoding pendrin protein have been shown to be associated with PDS. It has been recently demonstrated that some families with features of PDS do not have the inner ear malformations and mutations in the PDS gene. This condition has been named as “pseudo−Pendred syndrome” (pseudo−PDS), and has been hypothesized to be of autoimmune origin. Here we report four siblings who have goiter, severe hypothyroidism, a positive perchlorate discharge test and sensorineural deafness, but not the inner ear abnormality which is diagnostic for PDS. We suggest that thyroid peroxidase (TPO) gene should be analyzed in pseudo−PDS patients with congenital goitrous hypothyroidism and deafness.

**Conflict of interest:**None declared.

## INTRODUCTION

Pendred syndrome (PDS) is an autosomal recessive disorder first described in 1896 as a combination of congenital deafness and goiter (1). The iodide organification defect in PDS was revealed by perchlorate discharge test in 1958 (2). Despite the impaired iodide organification, most individuals with PDS are clinically and biochemically euthyroid (3). However, mild hypothyroidism may occur during childhood (4). The deafness in PDS is not caused by hypothyroidism (3), and is associated with inner ear abnormalities ranging from an isolated enlarged vestibular aqueduct to a cochlear malformation known as Mondini dysplasia (5, 6). These malformations of the inner ear that are essential for the diagnosis of true PDS can be detected by high−resolution computed tomography (CT) or magnetic resonance imaging (MRI) (7, 8).

The gene, being referred as PDS or SLC26A4, has been mapped to chromosome 7q in 1996 and encodes pendrin protein that functions as a transmembrane anion transporter in the thyroid and in the inner ear (9, 10). To date, more than 100 mutations of the PDS gene have been shown to be associated with PDS (11). However, it has been recently demonstrated that some families with features of PDS do not have the inner ear malformations or mutations in the PDS gene. This condition has been named as “pseudo− Pendred syndrome” (pseudo−PDS), and has been hypothesized to be of autoimmune origin (12, 13). On the other hand, some case reports have shown an association between congenital goitrous hypothyroidism and sensorineural deafness with mutations in the thyroid peroxidase (TPO) gene (14, 15). Accordingly, a TPO defect along with hearing impairment might be considered in the etiology of pseudo−PDS.

Here we report four siblings who have goiter, severe hypothyroidism, positive perchlorate test and sensorineural deafness, but not the inner ear abnormality which is diagnostic for PDS.

## CASE REPORT

**Figure 1:**
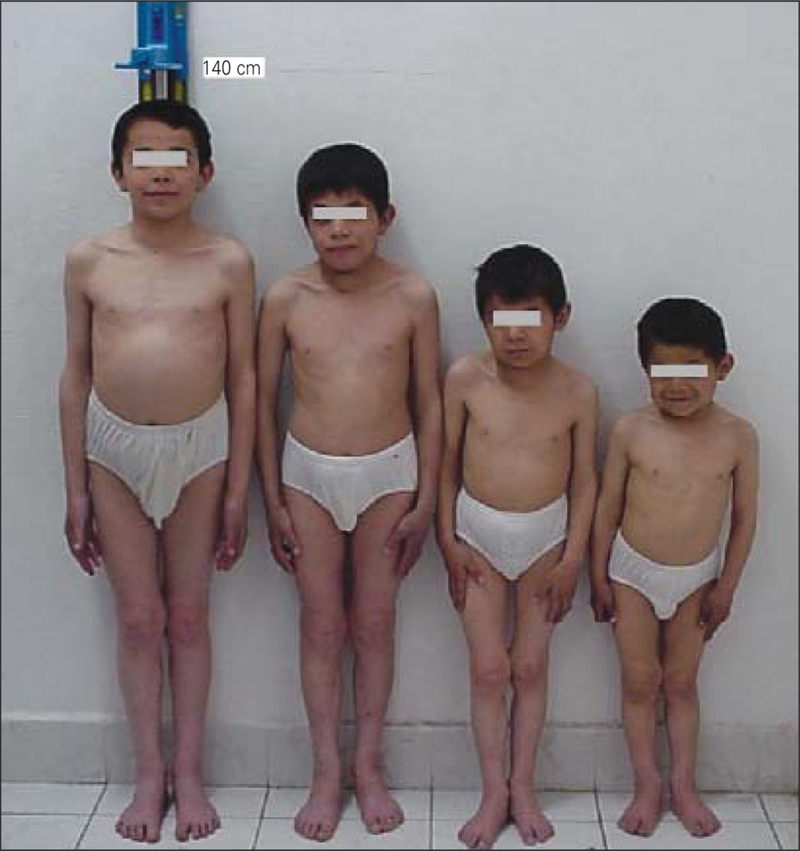
Four siblings with severe short stature caused by congenital goitrous hypothyroidism

Four siblings presented with main complaints of short stature and deaf−mutism at ages ranging between 10.5 and 17.3 yrs (Figure 1). Their parents were cousins, and had no signs of hypothyroidism, goiter or hearing loss. Besides congenital deafness, the patients had mental and developmental delay. We could not measure their mental levels, because they could not speak. Their growth had also been retarded since birth. On physical examination, all four children had multinodular goiter and severe short stature. Their height SD scores were between −5.1 SD and −7.3 SD, and their bone ages were severely retarded. The clinical and laboratory data of the siblings are summarized in Table 1.

Ultrasound findings, which were similar in all siblings, consisted of enlarged thyroid glands (volumes ranging from 12 ml to 42 ml) with multiple milimetric nodules and normal echo patterns. Free thyroxine (fT4) and thyrotropin (TSH) levels were compatible with severe hypothyroidism (Table 1). Anti−TPO and anti−thyroglobulin antibodies were negative in all siblings. The perchlorate tests, performed in three of the patients showed 131I discharges ranging between 51.9% and 75.0% (normal <10%), indicating the complete defect in iodide organification into thyroglobulin. L−thyroxine replacement therapy was initiated to treat the congenital goitrous hypothyroidism caused by iodide organification defect.

Audiological examination revealed bilateral profound sensorineural hearing loss (pure tone audiometry >80 dB) in all four siblings. However, high−resolution CT of the temporal bones of the siblings did not show any cochlear malformation, such as enlarged vestibular aqueducts and dilated endolymphatic sacs, which are almost specific for PDS and prerequisite for its diagnosis. At this stage, we decided to perform a molecular genetic analysis for definite diagnosis, which could not be performed due to the unwillingness of the parents to give us permission to analyze the TPO and PDS genes. The family also failed to bring their children for follow−up.

**Table 1:**
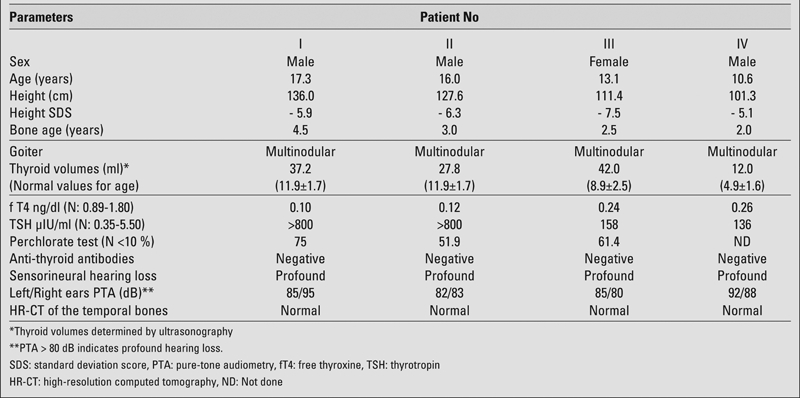
Clinical and laboratory data of the siblings

## DISCUSSION

According to the Fraser criteria (16), which include congenital deafness, goiter and a positive perchlorate discharge test, at first glance, our patients appear to be cases of PDS. However, some authors have advocated that inner ear abnormalities must be included as an essential prerequisite for the diagnosis (8, 17). Although a Mondini malformation of the cochlea was present only in a minority of patients (5), the enlargement of the endolymphatic duct and sac, and of the vestibular aqueduct have been documented by high−resolution CT and MRI in 80−100% of patients with PDS (7, 8). Fugazzola et al (12) studied the PDS gene mutations in nine patients from six families with a clinical diagnosis of PDS based on sensorineural hearing loss, multinodular goiter and a positive perchlorate test. They found that all seven patients, who were homozygous or compound heterozygous for PDS mutations, had the inner ear malformations detected by high−resolution CT or MRI; but, interestingly, the inner ear structures were normal in two sisters without mutations. The latter patients were referred to as pseudo−PDS by the authors. This important study has demonstrated that the differential diagnosis between PDS and pseudo−PDS should be based on the presence of inner ear malformations and, of course, of PDS gene mutations. Unfortunately, we could not do a molecular analysis. Yet, we performed high−resolution CT of temporal bones, but did not detect any inner ear abnormality in any of the four siblings. Thus, the finding of a normal inner ear was consistent with the diagnosis of pseudo−PDS, and not with that of PDS.

Moreover, while most cases of PDS are euthyroid, our patients had severe congenital hypothyroidism (CH) causing mental retardation, severe short stature, and remarkably delayed bone age. Some patients are reported to have subclinical or mild hypothyroidism that usually appears after mid−childhood (3, 4, 16, 17, 18). But one can still consider that these siblings are extremely rare examples of PDS manifested as severe CH. Reviewing the literature we found only one study on genetically confirmed PDS among patients with CH (19). In that study, out of 197 children with neonatal screening−detected CH, three fulfilled the clinical diagnostic criteria of PDS, and only two had the PDS gene mutations. Interestingly, these two true PDS patients had been diagnosed at birth as cases of subclinical and mild hypothyroidism, while a pseudo−PDS patient had presented with more severe CH (TSH: 150 mIU/L and T4: 1.16 μg/dl). This study indicates that PDS is a rare cause of CH, and also that PDS patients are mildly affected by CH. Therefore, since the severe state of hypothyroidism in our patients was not compatible with a diagnosis of PDS, we speculated that the condition in our patients should be explained by a reason other than PDS.

In the study by Fugazolla et al (12), the patients with PDS (age range:14−41 years) were identified to be cases of subclinical hypothyroidism with normal fT4 and TSH values, whereas two patients with a pseudo−PDS phenotype had overt hypothyroidism, probably caused by an autoimmune thyroid disease. Davis et al (13) reported dizygotic twins diagnosed to have sensorineural deafness and hypothyroidism caused by autoantibodies against the inner ear and thyroid tissues (anti−DEP−1/CD148 and anti−TPO, respectively), suggesting a link between autoimmune inner ear disease and autoimmune thyroid disease. The authors suggest that investigation for antibodies is warranted in children with possible PDS who are negative for PDS gene mutations and do not manifest the classical radiological findings. In addition, the perchlorate test is not necessarily conclusive of PDS, because positive results are reported in 20−80% of patients with Hashimoto thyroiditis (20). In our patients, we excluded the possibility of autoimmune damage to the thyroid gland since the results of antibody tests were negative and ultrasound examination of the thyroid gland revealed normal results.

In fact, the combination of CH, goiter and a positive perchlorate test indicating iodide organification defect should first remind one of the possibility of dyshormonogenesis due to a TPO defect (21). Although patients with TPO defect do not usually have deafness, some cases with both TPO defect and hearing loss, have been reported (14, 15). Pfarr et al (15) described a child who was clinically diagnosed to have PDS, but had only a monoallelic mutation in the PDS gene and a functionally normal pendrin protein. This patient, exhibiting a pseudo−PDS phenocopy, was compound heterozygous for mutations in the TPO gene. This study demonstrates that other genetic explanations for hearing loss should be considered in pseudo−PDS patients, and the TPO gene is the primary candidate gene in this instance. Because congenital deafness is very common, affecting 1 of 2000 newborns (18), with more than 50 different genes found to be involved in it (22), the origin of hearing loss in cases of pseudo−PDS may be due to mutations in other deafness genes.

In conclusion, although we were not able to confirm it by molecular analysis, we clinically diagnosed four siblings with congenital severe hypothyroidism due to impaired iodide organification and an accompanying congenital deafness owing to, probably, a TPO or other genetic defect. We also speculate that our patients with goiter and hearing loss may possibly be pseudo−PDS cases, because of their structurally normal inner ears, and their clinically and biochemically severe hypothyroid state. To establish the clinical diagnosis of PDS, imaging studies of temporal bones are mandatory and must be performed before genetic analysis. If the inner ear structures are normal, the patients should be initially investigated for anti−thyroid antibodies, and if required, for anti−cochlear antibodies. We also suggest that the TPO gene should be analyzed in pseudo−PDS patients with congenital goitrous hypothyroidism and deafness.
